# Nexus of grazing management with plant and soil properties in northern China grasslands

**DOI:** 10.1038/s41597-020-0375-0

**Published:** 2020-02-04

**Authors:** Li Wang, Limin Luan, Fujiang Hou, Kadambot H. M. Siddique

**Affiliations:** 1 Gansu Provincial Key Laboratory of Aridland Crop Science, Gansu Key Laboratory of Crop Genetics and Germplasm Enhancement, College of Life Science and Technology, Gansu Agricultural University, Lanzhou, 730070 China; 2 Agriculture and Agri-Food Canada, Swift Current Research and Development Centre, Swift Current, Saskatchewan Canada; 3 State Key Laboratory of Grassland Agro-ecosystems; Key Laboratory of Grassland Livestock Industry Innovation, Ministry of Agriculture and Rural Affairs; College of Pastoral Agriculture Science and Technology, Lanzhou University, Lanzhou, 730020 China; 4 The UWA Institute of Agriculture and School of Agriculture & Environment, The University of Western Australia, LB 5005, Perth, WA 6001 Australia

**Keywords:** Agroecology, Environmental impact, Sustainability

## Abstract

Grasslands provide habitats for living organisms and livelihoods for ~800 million people globally. Many grasslands in developing countries are severely degraded. Some measures have been taken to curb the trend of degradation for decades. It is important to determine how decade-long rejuvenation efforts affected grassland ecosystems. We identified 65 data-rich studies based on six criteria, from >2500 relevant publications, and generated a dataset with 997 rows and 12 variables. The dataset covers different grazing intensities (grazing exclusion, light, moderate, and heavy grazing) and their impacts on plant traits (vegetation coverage, aboveground and root biomass, and plant diversity) and soil physiochemical properties (bulk density, moisture content, organic C, total and available N, total and available P, C:N ratio, and pH). The dataset could be used to (i) quantify the effectiveness of rejuvenation processes by determining the impact on plant community and soil properties, (ii) perform comprehensive analyses to elucidate large-picture effects of grazing management and rejuvenation, and (iii) analyze the impact of grass–climate–soil–human interactions on grassland ecosystem sustainability.

## Abstract (summary)

**Table:** 

Measurement(s)	plant trait • structure of soil • composition of soil • soil biological activity
Technology Type(s)	digital curation
Factor Type(s)	grazing intensity (grazing exclusion, light, moderate, and heavy grazing)
Sample Characteristic - Environment	grassland ecosystem
Sample Characteristic - Location	China

Machine-accessible metadata file describing the reported data: 10.6084/m9.figshare.11663340

## Background & Summary

Grasslands cover ~50 million square kilometers or ~40 percent of the terrestrial area on Earth (excluding Greenland and Antarctica), and comprise various types including prairies, savannahs, rangelands, agricultural grasslands, and coastal grasslands^1^. As one of the largest ecosystems, grasslands are essential to living organisms including plants, animals and bird species, and it functions as a habitat for wildlife^2^ and livestock^3^, and the livelihoods of ~800 million people globally^4^.

China has the largest grasslands in the world with ~330 million hectares used for feeding animals for human foods^5^. However, a significant proportion has been degraded since the 1960s, mainly due to: (i) large-scale land reclamation from grasslands to croplands in the 1980s^6,7^ that aimed at producing more grain-based food for the growing human population but led to a rapid decline in available grasslands for grazing^8^ and accelerated the degradation of the remaining grasslands^9^; (ii) an expansion of the livestock industry in the 1990s that reduced the amount of grasslands per head of livestock^10^, and led to overgrazing of the remaining grasslands with high stocking rates^11^ and decreased grassland productivity^12,13^; (iii) exploitation of industrial by-products or mineral resources in some conventional grassland areas^14^ that decreased feeding-type grasslands; (iv) freshwater shortages for agriculture that presented a major problem for restoring grazing-induced degradation, especially in the arid and semiarid northwest where annual precipitation is typically <160 mm^15^ and annual evaporation >1,800 mm^16^; and (v) a shift from traditional nomadic grazing to sedentary feeding systems in the 1990s and 2000s that led to the loss of self-recovery capacity of grazed grasslands^17^ and reduced the intrinsic biological functions for soil structure^18^ and ecosystem equilibrium^19^.

To rejuvenate degraded grasslands, China took drastic measures by establishing a number of rejuvenation programs—including ‘grazing exclusion’ that aimed to eliminate grazing grasslands^17^, ‘grain-for-green’ program that returned steep cultivated land to grassland^20^ to alleviate the shortage of forage availability for the livestock industry^21^, ‘returning croplands to grasses’ that involved re-seeding grasses on marginal lands of cropping^22^—combined with ‘alternating seasonal-grazing with fallowing’ in moderately degraded grasslands^23^. Some of these programs have been in place for decades. Numerous studies have been conducted to determine the effectiveness of these programs for rejuvenating degraded grasslands; there is large variation between the studies and between geolocations, largely due to the variation in climatic conditions, grass types, duration of grazing practices, and the magnitude of human activities. It is often misleading to draw general conclusions from individual or site-specific experiments. A systematic analysis is required to define the outcome of the decade-long rejuvenation efforts, and a large dataset across various studies provides a unique opportunity to elucidate those effects.

**Fig. 1:**
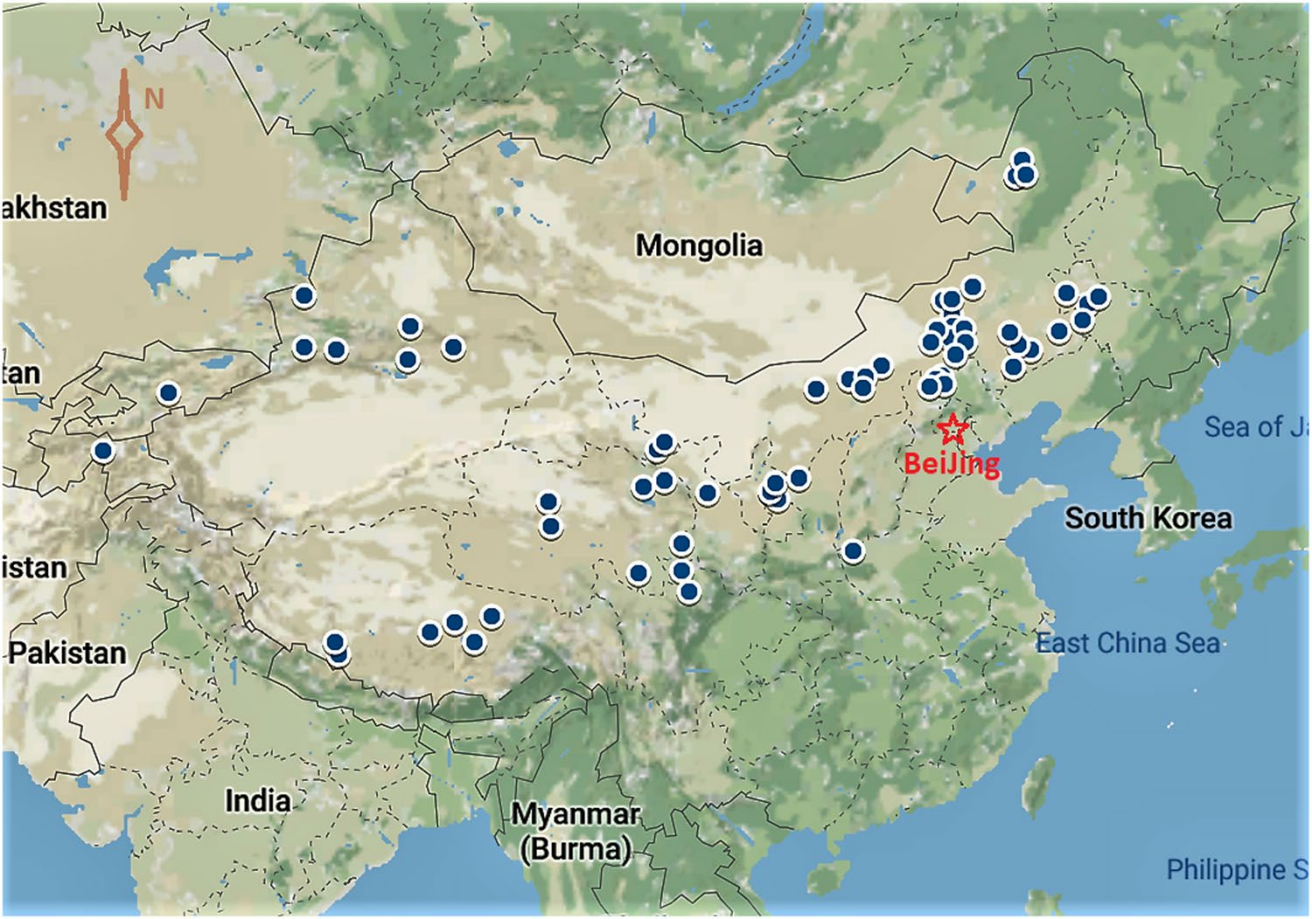
Geographic locations of the selected studies for the Data Descriptor. The selected locations/sites (indicated by blue dots) cover the major grassland areas in China from the semi-desert and arid northwest to semiarid and humid northeast regions.

We generated a comprehensive dataset^24^ derived from multiple studies on the relationship among grazing intensities, duration of grazing exclusion, and plant community traits and soil physiochemical properties. Data were extracted from 65 data-rich studies (Online-only Table 1) conducted across the major grassland areas in northern China (Fig. 1) with representative grassland types and sub-types included. The key variables include plant traits (percent vegetation coverage, plant biomass, root biomass, and plant diversity) and soil physiochemical properties (soil bulk density, moisture content, organic C, total N and available N, total and available P, C:N ratio, and soil pH).

We organized the dataset with a ‘Master’ tab and 15 associated tabs. This dataset can be used to (i) determine whether the decade-long grazing management policies have had positive, negative or neutral impacts on plant community composition and diversity, vegetation characteristics, and soil physiochemical properties; (ii) assess plant–soil–climate interactions using a systemic approach as the dataset contains key information on plants, soil, grassland ecosystems, and geographic information; and (iii) conduct comprehensive assessments of the effectiveness of grazing policies on ecological and socioeconomic implications. The synthesis of this dataset can help draw sound recommendations to manage grasslands sustainably and effectively.

## Methods

We employed a three-step approach for data collection and synthesis.

### First – literature search

**Fig. 2:**
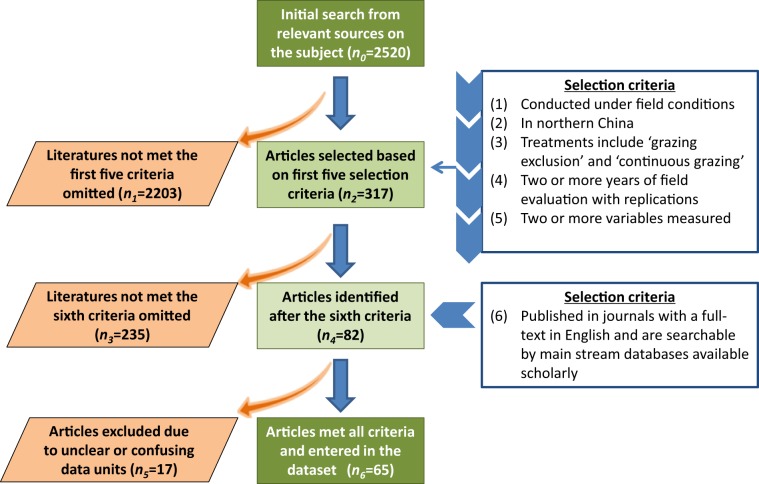
Workflow chart for generating dataset output. Brown boxes represent the number of articles (*n*_*i*_*, where i* = *0, 1… or 6*), included or excluded, step-by-step, based on the selection criteria; dark green boxes denote the articles selected for the present Data Descriptor. The six selection criteria are briefly described.

We conducted a comprehensive search of peer-reviewed literature, mainly through Agricola, Google Scholar, and Scopus. Search terms were defined and used to query the Institute for Scientific Information Web of Science since 1979, when the first grazing policy took place in China. The initial search in the article title, abstract and keywords revealed 2,520 articles (*n*_0_; Fig. 2) of potential interest. From the initial search, we identified articles meeting the first five criteria: (i) studies conducted under field conditions excluding those conducted in a controlled environment or simulation study; (ii) studies conducted in northern China; (iii) studies including ‘non-grazing or grazing exclusion’ and ‘continuous grazing’ treatments in their experimental structure with other treatments considered optional or additional; (iv) at least two years of field evaluation with replications each year; and (v) at least two or more variables measured. These criteria narrowed the selection of searched articles to 317 (*n*_2_; Fig. 2). We narrowed the 317 articles further to 65 using additional criteria—(vi) articles published in journals with a full-text in English and are searchable by main stream databases available scholarly, such as Scopus or Web of Science^25^. Non-English articles or articles that have an English abstract only but lack the measurements described in the above-described criteria were excluded.

### Second – data extraction

We extracted relevant data on a treatment-by-treatment basis from each of the identified articles (Online-only Table 1), entered the data in Excel spreadsheets, examined visually for usefulness, and combined them into a ‘Master’ data file. Some of the results presented in graphs in the original articles were converted into values using a graph-to-value conversion program (https://automeris.io/WebPlotDigitizer/). In some studies, where the results were aggregated, treatment means with a standard error of the mean were determined. Additionally, we reviewed 12 other articles that presented meta-analysis results, including articles describing the effects of grazing exclusion on soil C sequestration that included 78 studies^26^, plant biomass from 48 studies^27^, soil microbial communities from 71 studies^28^, soil C and N cycling from 115 studies^29^, grassland management and greenhouse gas emissions from 67 studies^30^, seasonal grazing and soil respiration from a 6-year study^31^, and grassland management and soil chemical properties in different climates from 105 studies^32^. Relevant data points from these articles were selectively entered into our ‘Master’ file, if they met the criteria we defined above.

### Third - database structure

The Excel ‘Master’ file contained all the data points^24^ extracted from the original articles, detailed in 997 rows and in various columns (Table 1), with column (A) as code, (B) author name (first author only) and publication year, (C) latitude/longitude coordinates of the study, (D) and (E) ecological region and locations (city or province), (F) year(s) in which the field experiment was conducted, (G) types of grasslands evaluated in the published study (some of the studies did not specify the type), (H) duration (number of years) of grazing treatments imposed, (I) type of animals involved in the study, (J) name of the variable and units reported, and (K) depths of soil sampled for soil physiochemical property measurements.

**Table 1:** Detailed names and descriptions for each column shown in the ‘Master’ Tab of the Excel file.

Column	Name	Description
A	Sort	Codes for data sorting
B	Reference	Authors and year of publication
C	Coordinate	Coordinates where the field experiment was conducted
D	Region category	Sampling sites sorted into four categories based on geographical location
E	Location	Location of the study, province, followed by country
F	Study period	Year(s) in which the field study was conducted
G	Type of grassland	Specific grass type in the study
H	Duration of exclusion	Number of years that grazing exclusion was imposed
I	Animal	Type of animal in the grazing treatments
J	Variable	Specific variable and the unit; unit conversion detailed in Tab named ‘Units’
K	Soil depth (cm)	Soil depth for sampling to measure soil attributes
L	No grazing (NG)	Grazing exclusion treatment in the grazing intensity study
M	*n1*	Number of replicates in the NG treatment
N	Light grazing (LG)	Light or mild grazing treatment in the grazing intensity study
O	*n2*	Number of replicates in the LG treatment
P	NG–LG	Difference in the values between the NG and LG treatments
Q	Moderate grazing (MG)	Moderate grazing treatment in the grazing intensity study
R	*n*3	Number of replicates in the MG treatment
S	NG–MG	The difference in the values between the NG and MG treatments
T	Heavy grazing (HG)	Heavy or overgrazing treatment in the grazing intensity study
U	*n*4	Number of replicates in the HG treatment
V	NG–HG	Difference in the values between the NG and HG treatments
W	Standard error	Standard error across the four grazing intensities; calculated from four means
X	Data source	Extracted either from the abstract, tables or converted from figures presented in the original articles

Values in columns (L) to (W) related to the four different levels of grazing intensities, including (L) ‘non-grazing or grazing exclusion’, (N) ‘light or mild grazing’, (Q) ‘moderate grazing’, and (T) ‘heavy grazing or overgrazing’. The values in these four columns (L, N, Q, and T) were either actual values extracted directly from the original articles or converted into the same unit across studies. The four adjacent columns (i.e., columns M, O, R and U) following each of the four grazing-intensity columns are the number of replications used in the specific study. To facilitate further analysis (by the authors of the present paper or others who might be interested in using this dataset), we calculated the differences between (P) ‘grazing exclusion’ and ‘light grazing’, (S) ‘grazing exclusion’ and ‘moderate grazing’, and (V) ‘grazing exclusion’ and ‘heavy or overgrazing’. Standard errors in column (W) referred to the variation among the four grazing intensity treatments. In column (X), we specified the data origin, if they were converted from a figure in the original articles.

## Data Records

Having entered all the data extracted from original articles into the ‘Master’ tab in the Excel file described above, we created 12 separate tabs to provide detailed information on the 12 key variables derived from the ‘Master’ file. The ‘Article’ tab lists the 65 selected data-rich articles, including the names of attributes for the article, each starting with the name of the first author, followed by the name of the journal and other attributes of the articles.

The 12 variable tabs provide potential data-users with much-needed convenience and may enhance the usefulness of the dataset^24^. The 12 tabs to the right present data on four plant-related and eight soil-related variables: (1) ‘Veget%’ is percent vegetation coverage (%), (2) ‘AbvBiom’ is aboveground plant biomass (g m^−2^), (3) ‘RootBiom’ is root biomass (g m^−2^), (4) ‘PltDiv’ is plant diversity, (5) ‘BulkD’ is soil bulk density, (6) ‘Soil-H2O’ is soil moisture content, (7) ‘SOC’, soil organic carbon at 0–15 cm depth, (8) ‘Soil-N’ is total soil-N at 0–60 cm depth, (9) ‘Avail-N’ is available soil-N including NH_4_^+^-N and NO_3_^−^-N at 0–60 cm depth, (10) ‘Soil-P’ is total and available soil-P at 0–40 cm depth, (11) ‘C-N Ratio’ is C:N ratio as reported in original articles (not calculated from this dataset), and (12) ‘Soil-pH’ is soil pH. We used a similar layout for each of the 12 variables, considering ease of use for data-users. Two final tabs (‘Units’ and ‘Note’) provide background information on the calculation of each variable’s units and the categories and observations.

## Technical Validation

**Fig. 3:**
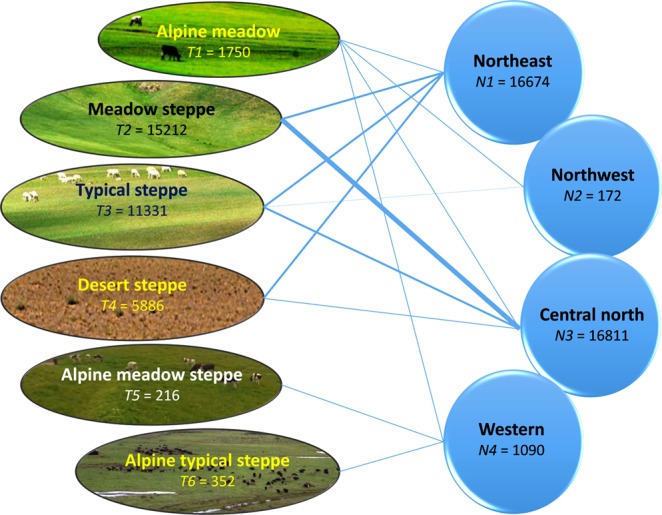
Six types of grasslands in the four ecological zones in China. T*i* (*where i* = *1, 2….0.6*) in each of the six types represents the number of treatments × studies × years; N*i* (*where i* = *1, 2….0.4*) in each of the four ecozones represents the number of treatments × studies × years.

Six grassland types (*Ti*, where *i* = 1, 2, 3, 4, 5, and 6) were included, with the number of observations varying among the four ecological zones (*Nj*…with *j* = 1, 2, 3, and 4) (Fig. 3). The dataset contained 34,747 observations by experimental site × growing season × treatments × grassland types.

**Fig. 4:**
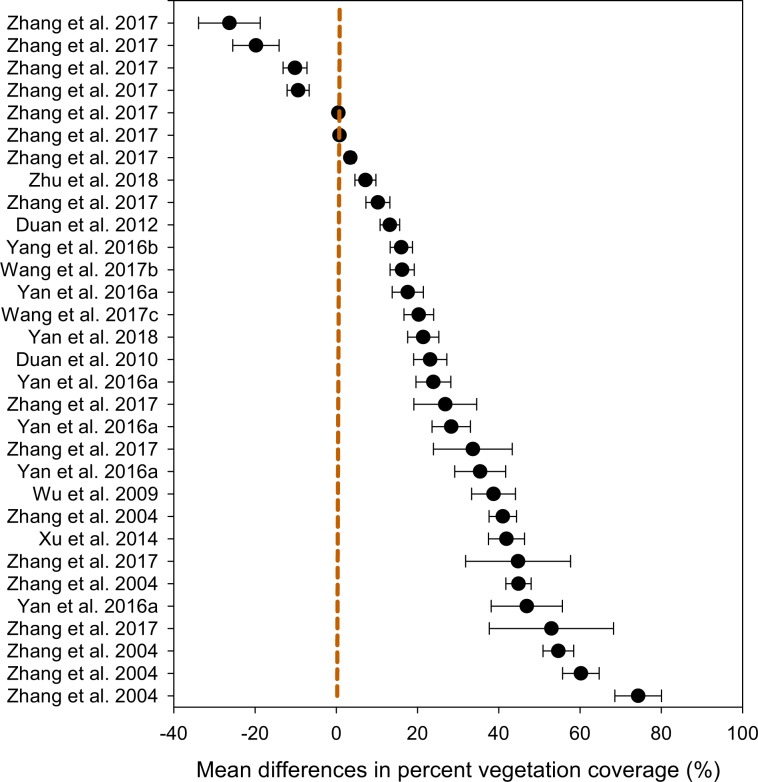
Distribution patterns in percent vegetation coverage between ‘grazing’ and ‘non-grazing’. Mean difference ≥0 means the results favor ‘non-grazing’, ≤0 means the results favor ‘grazing’, and a value of 0 means no difference between the two grazing practices. The distribution pattern shows the 95% confidence interval.

**Fig. 5:**
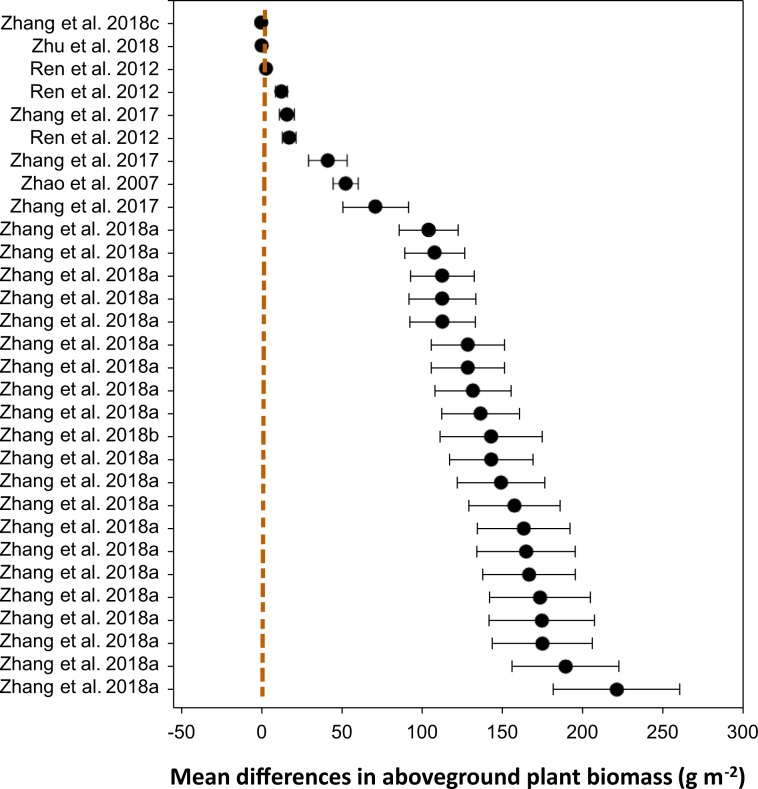
Distribution patterns in aboveground plant biomass between ‘grazing’ and ‘non-grazing’. Mean difference ≥0 means aboveground plant biomass yield favors ‘non-grazing’, ≤0 means the result favors ‘grazing’, and a value of 0 means no difference between the two practices. The distribution pattern shows the 95% confidence interval.

**Fig. 6:**
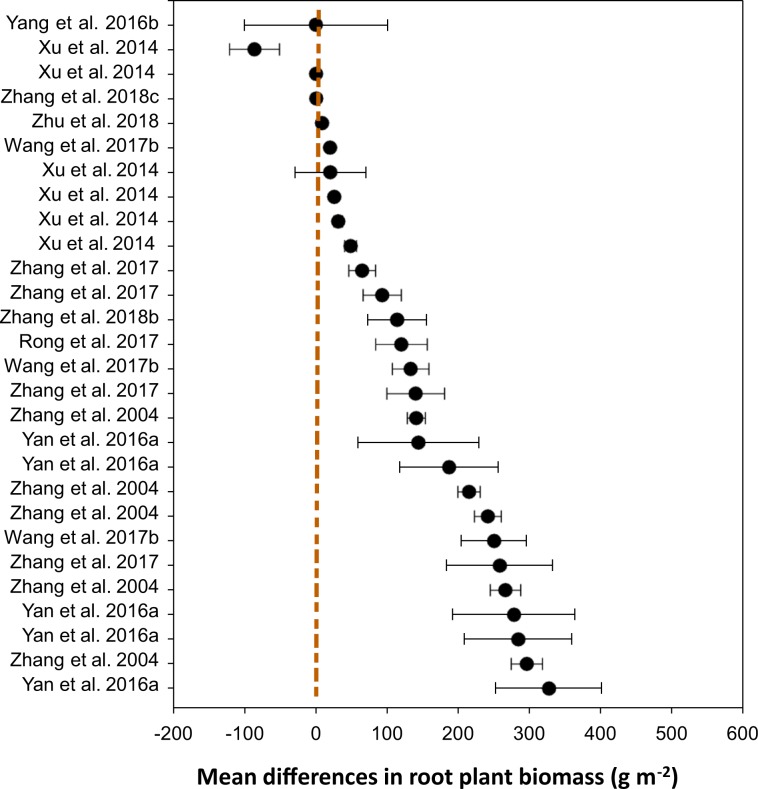
Distribution patterns in root biomass between ‘grazing’ and ‘non-grazing’. Mean difference ≥0 means root biomass yield favors ‘non-grazing’, ≤0 means the result favors ‘grazing’, and a value of 0 means no difference in root biomass between the two practices. The distribution pattern shows the 95% confidence interval.

**Fig. 7:**
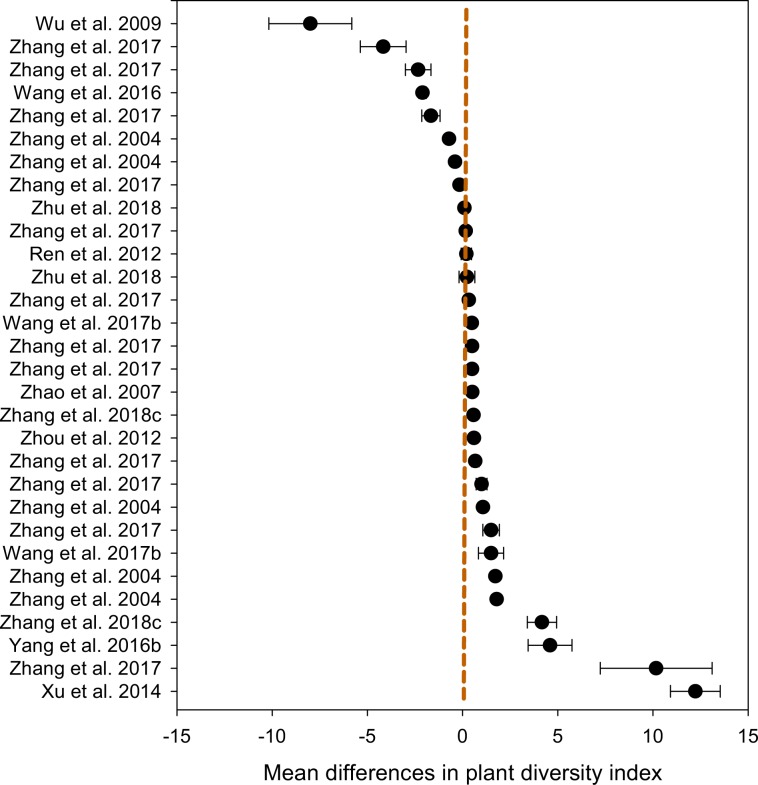
Distribution patterns in plant diversity between ‘grazing’ and ‘non-grazing’. Mean difference ≥0 means plant diversity favors ‘non-grazing’, ≤0 means the result favors ‘grazing’, and a value of 0 means no difference in plant diversity between the two practices. The distribution pattern shows the 95% confidence interval.

Each of the 12 key variables had enough data points to perform some basic analyses. To validate the usefulness of the dataset, we present four plant-related variables to demonstrate how the dataset could be analyzed by potential users, as follow:

For the variable ‘percent vegetation coverage’, we demonstrated that the mean difference (*n* = 226) between ‘grazing’ and ‘non-grazing’ practices was 20.61% (±1.43), ranging from 17.8 to 23.4% (Table 2). The distribution patterns across the studies showed that the mean differences between the two grazing systems for each of the studies (Fig. 4), where the majority of the studies showed a greater vegetation coverage under non-grazing practice compared to that under continuous grazing. Similarly, for the variable ‘aboveground plant biomass’, the mean difference between the two grazing practices was 6.67 (±0.84) kg ha^−1^, ranging from 5.02 to 8.31 kg ha^−1^ (Table 3). Apart from two studies that did not show a difference, all studies showed that aboveground plant biomass distribution patterns favored ‘non-grazing’(Fig. 5). Of the 32 studies that measured root biomass, 28 favored ‘non-grazing’ over ‘grazing’ with a mean difference in root biomass of 97.6 (±7.3) kg ha^−1^, ranging from 83.2 to 111.9 kg ha^−1^ (Table 4), and the distribution patterns favored the non-grazing practices with a few exception (Fig. 6). For the variable ‘plant diversity’, the effect of grazing practices was marginal, despite *p*-values > 0.001 (Table 5), and the distribution patterns scattered widely among studies (Fig. 7), where the mean differences in plant diversity varied considerably, with some studies skewed to the left, a few others skewed to the right, and the remainder with a mean difference near zero (i.e., the central vertical line). These distribution patterns are presented to validate the dataset only and illustrate how the dataset could be used by potential users.

**Table 2:** Percent vegetation coverage between grazing and non-grazing practices in northern China grasslands.

Study and effect	Duration^†^	Statistics						
		Difference in means	Standard error	Variance	Lower limit	Upper limit	*Z*-value	*p*-value
	(# of yrs)	----------------------------------- (%) ------------------------------------						
Zhang *et al*. 2017	7	−26.3	7.6	57.8	−41.2	−11.4	−3.5	0.001
Zhang *et al*. 2017	6	−19.8	5.7	32.7	−31.0	−8.6	−3.5	0.001
Zhang *et al*. 2017	1	−10.2	2.9	8.6	−15.9	−4.4	−3.5	0.001
Zhang *et al*. 2017	10	−9.4	2.7	7.4	−14.7	−4.1	−3.5	0.001
Zhang *et al*. 2017	4	0.5	0.1	0.0	0.2	0.8	3.5	0.001
Zhang *et al*. 2017	3	0.8	0.2	0.0	0.3	1.2	3.5	0.001
Zhang *et al*. 2017	12	3.4	1.0	1.0	1.5	5.4	3.5	0.001
Zhu *et al*. 2018	1	7.1	2.6	6.6	2.1	12.2	2.8	0.006
Zhang *et al*. 2017	9	10.2	3.0	8.7	4.4	16.0	3.5	0.001
Duan *et al*. 2012	4	13.2	2.4	5.9	8.4	17.9	5.4	0.000
Yang *et al*. 2016b	5	16.0	2.7	7.5	10.6	21.4	5.8	0.000
Wang *et al*. 2017b	18	16.2	3.0	8.9	10.3	22.1	5.4	0.000
Yan *et al*. 2016a	5	17.6	3.8	14.6	10.1	25.1	4.6	0.000
Wang *et al*. 2017c	10	20.3	3.7	13.4	13.1	27.5	5.5	0.000
Yan *et al*. 2018	18	21.4	3.8	14.6	13.9	28.9	5.6	0.000
Duan *et al*. 2010	3	23.1	4.1	16.6	15.1	31.1	5.7	0.000
Yan *et al*. 2016a	4	23.9	4.3	18.4	15.5	32.3	5.6	0.000
Zhang *et al*. 2017	11	26.8	7.7	60.0	11.6	42.0	3.5	0.001
Yan *et al*. 2016a	3	28.3	4.7	22.3	19.0	37.6	6.0	0.000
Zhang *et al*. 2017	8	33.6	9.7	94.1	14.6	52.6	3.5	0.001
Yan *et al*. 2016a	2	35.4	6.3	39.2	23.1	47.7	5.7	0.000
Wu *et al*. 2009	10	38.7	5.4	29.1	28.1	49.3	7.2	0.000
Zhang *et al*. 2004	1	41.0	3.4	11.4	34.4	47.6	12.1	0.000
Xu *et al*. 2014	11	41.9	4.5	19.8	33.2	50.6	9.4	0.000
Zhang *et al*. 2017	5	44.8	12.9	166.9	19.4	70.1	3.5	0.001
Zhang *et al*. 2004	2	44.8	3.1	9.6	38.8	50.9	14.5	0.000
Yan *et al*. 2016a	1	46.9	8.8	76.7	29.7	64.1	5.4	0.000
Zhang *et al*. 2017	2	53.0	15.3	233.6	23.0	82.9	3.5	0.001
Zhang *et al*. 2004	4	54.7	3.8	14.3	47.3	62.1	14.5	0.000
Zhang *et al*. 2004	3	60.2	4.5	20.3	51.4	69.0	13.4	0.000
Zhang *et al*. 2004	5	74.3	5.7	32.9	63.1	85.5	13.0	0.000
Mean (*n* = 226)	6.1	20.61	1.43	2.04	17.81	23.41	14.42	0.000

^†^The duration (number of years) of the treatments (Master tab^24^) or the number of samplings for the specific study.

**Table 3:** Aboveground plant biomass between grazing and non-grazing practices in northern China grasslands.

Study and effect	Duration^†^	Statistics						
		Difference in means	Standard error	Variance	Lower limit	Upper limit	*Z*-value	*p*-value
	(# of yrs)	----------------------------------- (g m^−2^) --------------------------------						
Zhang *et al*. 2018c	11	0.1	0.0	0.0	0.1	0.1	5.5	0.000
Zhu *et al*. 2018	1	0.4	0.1	0.0	0.2	0.6	5.1	0.000
Ren *et al*. 2012	25	3.1	0.6	0.4	1.9	4.3	5.1	0.000
Ren *et al*. 2012	9	12.7	3.7	13.9	5.4	20.0	3.4	0.001
Zhang *et al*. 2017	25	16.1	4.6	21.5	7.0	25.1	3.5	0.001
Ren *et al*. 2012	9	17.5	4.2	18.0	9.2	25.8	4.1	0.000
Zhang *et al*. 2017	5	41.5	12.0	143.4	18.0	64.9	3.5	0.001
Zhao *et al*. 2007	25	52.6	7.8	60.3	37.4	67.8	6.8	0.000
Zhang *et al*. 2017	9	71.0	20.5	420.2	30.8	111.2	3.5	0.001
Zhang *et al*. 2018a	2	104.0	18.4	339.1	68.0	140.1	5.7	0.000
Zhang *et al*. 2018a	2	107.9	18.7	350.2	71.2	144.5	5.8	0.000
Zhang *et al*. 2018a	2	112.6	19.9	394.3	73.7	151.5	5.7	0.000
Zhang *et al*. 2018a	2	112.6	20.8	434.2	71.8	153.5	5.4	0.000
Zhang *et al*. 2018a	2	112.7	20.4	416.7	72.7	152.7	5.5	0.000
Zhang *et al*. 2018a	2	128.5	22.8	519.2	83.8	173.1	5.6	0.000
Zhang *et al*. 2018a	2	128.5	22.8	520.2	83.8	173.2	5.6	0.000
Zhang *et al*. 2018a	2	131.7	23.8	564.7	85.1	178.2	5.5	0.000
Zhang *et al*. 2018a	2	136.4	24.3	588.7	88.9	184.0	5.6	0.000
Zhang *et al*. 2018b	2	143.0	31.9	1016.1	80.5	205.5	4.5	0.000
Zhang *et al*. 2018a	2	143.1	26.0	675.4	92.1	194.0	5.5	0.000
Zhang *et al*. 2018a	2	149.1	27.3	745.6	95.6	202.6	5.5	0.000
Zhang *et al*. 2018a	2	157.5	28.4	808.9	101.8	213.3	5.5	0.000
Zhang *et al*. 2018a	2	163.3	28.9	833.1	106.7	219.9	5.7	0.000
Zhang *et al*. 2018a	2	164.7	30.7	940.7	104.6	224.9	5.4	0.000
Zhang *et al*. 2018a	2	166.6	28.9	833.2	110.0	223.2	5.8	0.000
Zhang *et al*. 2018a	2	173.4	31.4	986.4	111.9	235.0	5.5	0.000
Zhang *et al*. 2018a	9	174.5	32.9	1080.4	110.1	238.9	5.3	0.000
Zhang *et al*. 2018a	2	174.9	31.2	972.9	113.7	236.0	5.6	0.000
Zhang *et al*. 2018a	2	189.3	33.3	1108.5	124.1	254.6	5.7	0.000
Zhang *et al*. 2018a	2	221.1	39.4	1551.7	143.9	298.3	5.6	0.000
Mean (*n* = 232)		6.67	0.84	0.70	5.02	8.31	7.94	0.000

^†^The duration (number of years) of the treatments (Master tab^24^) or the number of samplings for the specific study.

**Table 4:** Root biomass between grazing and non-grazing practices in northern China grasslands.

Study and effect	Duration^†^	Statistics						
		Difference in means	Standard error	Variance	Lower limit	Upper limit	*Z*-value	*p*-value
	(# of yrs)	---------------------------------- (g m^−2^) ---------------------------------						
Yang *et al*. 2016b	5	−468.0	100.6	10124.9	−665.2	−270.8	−4.7	0.000
Xu *et al*. 2014	11	−86.0	35.0	1225.3	−154.6	−17.3	−2.5	0.014
Xu *et al*. 2014	11	0.4	2.7	7.3	−4.9	5.7	0.1	0.885
Zhang *et al*. 2018c	11	0.6	0.1	0.0	0.4	0.8	5.9	0.000
Zhu *et al*. 2018	11	8.7	1.6	2.5	5.6	11.8	5.5	0.000
Wang *et al*. 2017b	9	20.0	3.9	14.9	12.4	27.6	5.2	0.000
Xu *et al*. 2014	11	20.3	49.8	2480.8	−77.3	118.0	0.4	0.683
Xu *et al*. 2014	18	25.8	4.0	15.8	18.0	33.6	6.5	0.000
Xu *et al*. 2014	11	31.4	6.1	36.7	19.5	43.2	5.2	0.000
Xu *et al*. 2014	1	48.7	8.4	70.1	32.3	65.1	5.8	0.000
Zhang *et al*. 2017	4	65.0	18.8	352.1	28.2	101.8	3.5	0.001
Zhang *et al*. 2017	25	93.0	26.8	720.8	40.4	145.6	3.5	0.001
Zhang *et al*. 2018b	1	114.1	41.4	1713.4	33.0	195.2	2.8	0.006
Rong *et al*. 2017	2	120.0	36.0	1298.1	49.4	190.6	3.3	0.001
Wang *et al*. 2017b	25	133.0	25.6	655.5	82.8	183.2	5.2	0.000
Zhang *et al*. 2017	4	140.0	40.4	1633.3	60.8	219.2	3.5	0.001
Zhang *et al*. 2004	25	140.8	12.6	157.6	116.2	165.5	11.2	0.000
Yan *et al*. 2016a	3	144.0	85.1	7246.4	−22.8	310.8	1.7	0.091
Yan *et al*. 2016a	4	187.0	69.2	4783.0	51.4	322.6	2.7	0.007
Zhang *et al*. 2004	2	214.8	15.6	241.8	184.3	245.3	13.8	0.000
Zhang *et al*. 2004	5	241.2	18.7	349.9	204.5	277.9	12.9	0.000
Wang *et al*. 2017b	25	250.0	45.8	2093.3	160.3	339.7	5.5	0.000
Zhang *et al*. 2017	18	258.0	74.5	5547.0	112.0	404.0	3.5	0.001
Zhang *et al*. 2004	1	265.8	21.3	452.2	224.2	307.5	12.5	0.000
Yan *et al*. 2016a	3	278.0	85.9	7379.0	109.6	446.4	3.2	0.001
Yan *et al*. 2016a	5	284.0	75.5	5702.2	136.0	432.0	3.8	0.000
Zhang *et al*. 2004	18	295.8	21.9	480.5	252.8	338.7	13.5	0.000
Yan *et al*. 2016a	18	327.0	74.5	5549.0	181.0	473.0	4.4	0.000
Wang *et al*. 2017b	11	407.0	74.3	5527.5	261.3	552.7	5.5	0.000
Mean (*n* = 222)		97.6	7.3	53.6	83.2	111.9	13.3	0.000

^†^The duration (number of years) of the treatments (Master tab^24^) or the number of samplings for the specific study.

**Table 5:** Plant diversity between grazing and non-grazing practices in northern China grasslands.

Study and effect	Duration^†^	Statistics						
		Difference in means	Standard error	Variance	Lower limit	Upper limit	*Z*-value	*p*-value
	(# of yrs)	--------------------------- (diversity index) -----------------------------						
Wu *et al*. 2009	10	−8.00	2.18	4.74	−12.27	−3.73	−3.67	0.000
Zhang *et al*. 2017	25	−4.17	1.20	1.45	−6.53	−1.81	−3.46	0.001
Zhang *et al*. 2017	25	−2.33	0.67	0.45	−3.65	−1.01	−3.46	0.001
Wang *et al*. 2016	3	−2.10	0.19	0.04	−2.48	−1.72	−10.87	0.000
Zhang *et al*. 2017	25	−1.66	0.48	0.23	−2.60	−0.72	−3.46	0.001
Zhang *et al*. 2004	2	−0.71	0.06	0.00	−0.83	−0.58	−10.92	0.000
Zhang *et al*. 2004	1	−0.40	0.05	0.00	−0.49	−0.30	−7.98	0.000
Zhang *et al*. 2017	25	−0.16	0.05	0.00	−0.25	−0.07	−3.46	0.001
Zhu *et al*. 2018	1	0.10	0.06	0.00	−0.01	0.21	1.73	0.084
Zhang *et al*. 2017	25	0.17	0.05	0.00	0.07	0.27	3.46	0.001
Ren *et al*. 2012	9	0.20	0.27	0.07	−0.33	0.73	0.73	0.464
Zhu *et al*. 2018	2	0.23	0.42	0.17	−0.59	1.05	0.55	0.582
Zhang *et al*. 2017	25	0.33	0.10	0.01	0.14	0.52	3.46	0.001
Wang *et al*. 2017b	18	0.49	0.11	0.01	0.27	0.71	4.35	0.000
Zhang *et al*. 2017	25	0.50	0.14	0.02	0.22	0.78	3.46	0.001
Zhang *et al*. 2017	25	0.50	0.14	0.02	0.22	0.78	3.46	0.001
Zhao *et al*. 2007	5	0.51	0.08	0.01	0.35	0.67	6.09	0.000
Zhang *et al*. 2018c	11	0.58	0.10	0.01	0.38	0.78	5.80	0.000
Zhou *et al*. 2012	N/A	0.60	0.09	0.01	0.42	0.77	6.73	0.000
Zhang *et al*. 2017	25	0.67	0.19	0.04	0.29	1.05	3.46	0.001
Zhang *et al*. 2017	25	1.00	0.29	0.08	0.43	1.57	3.46	0.001
Zhang *et al*. 2004	3	1.07	0.08	0.01	0.91	1.24	12.74	0.000
Zhang *et al*. 2017	25	1.50	0.43	0.19	0.65	2.35	3.46	0.001
Wang *et al*. 2017b	17	1.50	0.66	0.44	0.21	2.79	2.27	0.023
Zhang *et al*. 2004	4	1.72	0.15	0.02	1.43	2.02	11.30	0.000
Zhang *et al*. 2004	5	1.80	0.14	0.02	1.52	2.07	12.90	0.000
Zhang *et al*. 2018c	10	4.17	0.77	0.59	2.66	5.68	5.41	0.000
Yang *et al*. 2016b	5	4.60	1.15	1.33	2.34	6.86	3.99	0.000
Zhang *et al*. 2017	25	10.17	2.94	8.62	4.42	15.92	3.46	0.001
Xu *et al*. 2014	11	12.23	1.31	1.71	9.67	14.79	9.36	0.000
Mean (*n* = 286)		0.47	0.14	0.02	0.19	0.74	3.34	0.001

^†^The duration (number of years) of the treatments (Master tab^24^) or the number of samplings for the specific study.

## Usage Notes

The compilation of experimental field data from 65 data-rich studies resulted in a dataset full of meaningful information^24^, with a set of variables that cover the key attributes of grassland properties. We suggest that potential dataset-users can perform quantitative assessments on whether grassland rejuvenation processes have had a significant impact on grassland ecosystem properties.

In particular, the dataset could be used for the following:

1. To quantify whether rejuvenation programs have had a significant impact on aboveground plant communities and belowground soil properties by comparing grazing exclusion with other grazing practices (light/mild grazing, and year-around continuous grazing). The aboveground plant community variables—including percent vegetation coverage, quantity of aboveground and belowground biomass (primarily roots), and the derived ratio of aboveground to belowground biomass—can be used by modelers to estimate carbon input into soils by different plant parts. It also provides valuable information on the magnitude of plant diversity in relation to the diverse grassland rejuvenation practices^33^. Further, the dataset can be analyzed to assess whether the rejuvenation programs have had an impact on soil physiochemical properties, including soil bulk density, moisture content, organic carbon, total and available N, total and available P, soil C:N ratio, and soil pH. Such analyses can help to determine the degree of grassland degradation and rejuvenation and their impact on grassland ecosystem sustainability^33–36^. Additionally, the dataset includes a variable—duration (the number of years) of grazing practices—that distinguishes grazing exclusion from continuous grazing. A comparison of the two contrasting practices against the duration of grazing can help identify the trend of the effect in grassland properties. The latter feature is unique for this dataset, and is rarely found in existing scientific literature.
2. The dataset can be used as a solid base for performing more comprehensive analyses such as a meta-analysis^37^. The dataset presents the results from more than 65 well-designed field studies with two or more replicates and site-years. Analyzing the dataset using meta-analysis may help to elucidate large-picture effects. More data with treatment structure and measurements meeting the criteria defined above could be entered into the Master file to build an even stronger dataset for comprehensive analysis.
3. The dataset can be analyzed to learn the magnitude of grass–climate–soil–human interactions on the outcome of grassland degradation and rejuvenation. The collected data come from a wide range of grasslands spread across diverse landscapes from semi-desert, arid, semiarid to humid climatic zones with varying weather conditions. Climatic variability across grassland zones may have had an impact on some aspects of grassland properties such as species composition^38^, herb abundance^39^, and shrub encroachment^40^, as well as belowground properties^41^. Such analysis may help with understanding the complex nature of interactions among meteorological, topographic, and soil environments with plant community structures and management practices. This type of analysis may have significant societal value for policymakers, researchers, and the general public.

## Online-only Table

**Online-only Table 1:** Key variables extracted from various studies. The numbers in the table body refer to the references cited in the paper.

Coordinate	Study year	Duration (yr)^†^	Plant community				Soil properties								
							Physical			Chemical					Biolog.
			Biomass	Diversity	Veg. %	Root biom.	Bulk density	Texture	Moisture	SOC	N	Other nutrients	pH	C/N	
37°8′ N, 106°49′ E	2001–2014	13								^42^	^42^	^42^			^42^
37°8′ N, 106°49′ E	2001–2014	13													^43^
33°37′ N, 99°48′ E	1998–2000	2					^44^			^44^	^44^	^44^		^44^	
30°27′–35°39′ N, 83°41′–95°10′ E	2006–2009	3	^45^	^45^	^45^										
30°27′–35°39′ N, 83°41′–95°11′ E	2006–2010	N/A	^45^		^45^										
43°38′ N, 116°42′ E	2007–2009	N/A					^46^	^46^		^46^					
42°53′ N, 83°42′ E	N/A	27	^47^		^47^	^47^	^47^						^47^		
37°7′ N, 106°49′ E	2001–2012	11													^48^
43°34′ N, 119°35′–119°38′ E	2003–2005	N/A	^49^		^49^					^49^	^49^				
37°6′ N, 103°31′ E	2003–2010	7					^50^	^50^	^50^			^50^	^50^	^50^	^50^
43°33′ N, 116°40′ E	2004–2006	25					^51^	^51^		^51^	^51^				
41°46′–41°50′ N, 111°2′–111°55′ E	2005	8					^52^			^52^	^52^	^52^	^52^		
37°8′ N, 106°50′ E	2001–2009	8							^53^		^53^				^53^
37°8′ N, 106°50′ E	2001–2010	9	^54^							^54^	^54^		^54^	^54^	
44°33′ N, 123°40′ E	2009–2012	3							^55^	^55^	^55^	^55^	^55^	^55^	^55^
43°38′ N, 116°42′ E	2005–2010	9	^56^	^56^											
41°44′ N, 115°46′ E	2010–2014	4	^57^			^57^	^57^			^57^	^57^		^57^		
37°37′ N, 101°12′ E	2006–2009	4							^58^	^58^	^58^				^58^
43°38′ N, 116°42′ E	2005–2008	3	^59^							^59^					
43°38′ N, 116°42′ E	1979–2004	25						^60^		^60^	^60^		^60^		
30°57′ N, 88°42′ E	2012	2					^61^			^61^	^61^	^61^			
41°54′ N, 116°0′ E	2009–2010	1			^8^		^8^		^8^		^8^	^8^	^8^	^8^	
44°45′ N, 123°45′ E	N/A	5					^62^		^62^				^62^		
29°56′–36°41′ N, 83°52′–95°1′ E	1980–2007	27					^63^	^63^		^63^	^63^	^63^	^63^	^63^	
41°44′ N, 115°46′ E	2009–2012	3	^64^	^64^			^64^	^64^	^64^	^64^	^64^		^64^		
44°35′ N, 123°36′ E	2002–2003	17	^65^						^65^						
38°54′–39°11′ N, 100°48′–101°12′ E	2016	18	^66^	^66^	^66^	^66^	^66^			^66^	^66^		^66^	^66^	
43°38′ N, 116°42′ E	2005–2008	3					^9^			^9^	^9^	^9^			
33°42′ N, 102°7′ E	1999–2009	10	^67^	^67^	^67^										
43°38′ N, 116°42′ E	N/A	25													^68^
43°50′ N, 116°34′ E	1989–2006	15	^69^				^69^			^69^	^69^				
41°46′ N, 115°41′ E	2001–2012	11	^70^	^70^	^70^	^70^	^70^		^70^	^70^	^70^			^70^	
31°38′ N, 92°0′ E	2013	3							^71^		^71^	^71^	^71^		^71^
49°19′–49°20′ N, 119°56′–119°57′ E	2009–2014	5	^72^		^72^	^72^									
49°23′–49°25′ N, 120°5′–120°11′ E	2009–2014	5					^73^		^73^						
44°48′–44°50′ N, 116°2′–116°30′ E	2011–2012	N/A	^74^		^74^						^74^				
34°54′ N, 102°6′ E	2010–2015	5	^75^	^75^	^75^	^75^	^75^				^75^	^75^	^75^		
34°36′–35°53′ N, 111°15′–112°37′ E	N/A	N/A									^76^	^76^	^76^		
42°55′ N, 120°42′ E	1992–1996	5	^77^	^77^	^77^	^77^									
31°23′ N, 90°2′ E	2010–2013	3	^78^		^78^										
42°55′N, 120°42′E	2014	25	^79^	^79^	^79^	^79^	^79^		^79^		^79^		^79^		
43°50′ N, 87°37′ E	2004–2005	5	^80^	^80^											
42°52′ 43°57′ N, 83°42′–89°45′ E	2003	N/A		^81^											

^**†**^Number of years that the grazing exclusion practice was imposed.
